# Two new species of *Ateuchus* with remarks on ecology, distributions, and evolutionary relationships (Coleoptera, Scarabaeidae, Scarabaeinae)

**DOI:** 10.3897/zookeys.747.22731

**Published:** 2018-03-29

**Authors:** Victor Moctezuma, José Luis Sánchez-Huerta, Gonzalo Halffter

**Affiliations:** 1 Instituto de Ecología, A.C., Carretera antigua a Coatepec 351, El Haya, Xalapa, Veracruz, 91070, Mexico

**Keywords:** Ateuchini, distribution patterns, dung beetles, Los Chimalapas, Mexican Transition Zone, Mountain Mesoamerican, Ateuchini, escarabajos del estiércol, Los Chimalapas, Mesoamericano de Montaña, patrones de distribución, Zona de Transición Mexicana

## Abstract

Two new species of the genus *Ateuchus* Weber are described from the region of Los Chimalapas, Oaxaca, Mexico: *A. benitojuarezi* **sp. n.** and *A. colossus* **sp. n.** A diagnosis for distinguishing these new species from the other species of this genus in North America is included. This paper is illustrated with pictures of the dorsal habitus and the male genitalia of the new species. The evolutionary relationships of the species are discussed, as well as their distribution and ecology. It is considered that the species of the genus *Ateuchus* present in North and Central America correspond to the Typical Neotropical and Mountain Mesoamerican distribution patterns.

## Abstract (es)

Describimos dos especies nuevas del género *Ateuchus* Weber, de la región de Los Chimalapas, Oaxaca, México: *A. benitojuarezi* **sp. n.** y *A. colossus* **sp. n.** Incluimos diagnosis para distinguir a estas especies nuevas del resto de especies del género presentes en Norteamérica. Presentamos ilustraciones del hábito dorsal y la genitalia masculina de las nuevas especies. Discutimos las relaciones evolutivas de las especies, así como también su distribución y ecología. Consideramos que las especies del género *Ateuchus* corresponden con los patrones de distribución Neotropical Típico y Mesoamericano de Montaña.

## Introduction

*Ateuchus* Weber is a dung beetle genus found in the New World. *Ateuchus* includes species of small to moderate size, oval and very convex in shape. These species normally have a bidentate clypeus, concealed scutellum, elytra with eight striae, mesosternon largely exposed, and protibia possessing three or four teeth. Most of the species present a certain degree of sexual dimorphism, since the males can present an expanded protibial spur and differences in the clypeal shape (Kohlmann 2003).

The greatest diversity of *Ateuchus* is concentrated in South America, where more than one hundred species are estimated to occur. However, the taxonomic status of several species that conform this genus is confusing and a review is required. Mexico is one of the countries where the species of the genus *Ateuchus* are best known: 14 are recorded from the country, *A. candezei* (Harold), *A. carolinae* Kohlmann, *A. chrysopyge* (Bates), *A. gershensoni* Kohlmann, *A. guatemalensis* (Bates), *A. halffteri* Kohlmann, *A. hornai* (Balthasar), *A. illaesum* (Harold), *A. klugi* (Harold), *A. laetitiae* Kohlmann, *A. perezvelai* Kohlmann, *A. rodriguezi* (Predhomme de Borre), *A. texanus* Robinson and *A. tuza* Kohlmann and Vaz-de-Mello (Kohlmann and Vaz-de-Mello in press). The objective of this article is to describe two new species of *Ateuchus* from Mexico and to discuss their possible evolutionary relationships, distributions, and ecology.

## Materials and methods

Abbreviations for the collections used in this work are as follows:

**CEMT** Seção de Entomologia da Coleção Zoológica, Departamento de Biologia e Zoologia, Universidade Federal de Mato Grosso, Cuiabá, Brazil

**CMNC** Canadian Museum of Nature, Gatineau, Quebec, Canada

**CNIN** Colección Nacional de Insectos, Instituto de Biología, Universidad Nacional Autónoma de México

**FSCA** Florida State Collection of Arthropods, Gainesville, FL, USA

**IEXA** Sección G Halffter, Colección Entomológica Miguel Ángel Morón Ríos, Instituto de Ecología, A. C., Xalapa, Veracruz, Mexico

**JLSHC** JL Sánchez-Huerta Collection, Xalapa, Veracruz, Mexico

**TAMU** Texas A&M University Insect Collection, TX, USA

**VMC** V Moctezuma Collection, Xalapa, Mexico

For this study, the phylogenetic species concept was used. The phylogenetic species concept is classified in two distinct versions according to Zachos (2016): the monophyly version and the diagnosability version. We follow the diagnosability version (Wheeler and Platnick 2000), which defines species as the smallest aggregation of populations or lineages that is diagnosable by a unique combination of character states. All type specimens bear determination labels printed on red acid-free paper. The internal sacs and aedeagi were prepared as outlined by Moctezuma et al. (2016) and we followed the sclerite nomenclature proposed by Marchisio and Zunino (2012). Male genitalia were stored in microvials with glycerol. Measurements and pictures were taken using a Leica Z16APOA stereomicroscope and the software of the manufacturer (z-stack image capture method).

## Taxonomy

### Ateuchus benitojuarezi sp. n.

Taxon classificationAnimaliaColeopteraScarabaeidae

http://zoobank.org/AAE3B380-C535-4C1D-A3D0-6EDC82170386

Figs 1 , 2

#### Type material

(**100** ♂♂, **161** ♀♀). Holotype: 1 ♂, labeled “MEXICO, Oaxaca, San Miguel Chimalapa, San Antonio, 14-X-2015, coprotrap; 16°39'39.3"N, 94°13'23.6"W, cloud forest, 1605 m, V. Moctezuma col.”. Paratypes: 1 ♂, 1 ♀, same data as holotype; 1 ♂, 1 ♀, same data except “16°39'40.9"N, 94°13'25.1"W, 1611 m”; 1 ♂, same data except “16°3'38.3"N, 94°13'39.8"W, 1702 m”; 5 ♂♂, 7 ♀♀, same data except “16°39'42.5"N, 94°13'15.1"W, 1553 m”; 4 ♂♂, 8 ♀♀, same data except “16°39'41.2"N, 94°13'15.6"W, 1562 m”; 6 ♀♀, same data except “16°39'40.7"N, 94°13'19.7"W, 1593 m”; 3 ♂♂, 1 ♀, same data except “16°39'40.2"N, 94°13'21"W, 1595 m”; 1 ♂, 2 ♀♀, same data except “16°39'41.8"N, 94°13'13.9"W, 1548 m”; 1 ♀, same data except “16°39'37.5"N, 94°13'27.1"W, 1621 m”; 2 ♂♂, 3 ♀♀, same data except “16°39'41.1"N, 94°13'16.9"W, 1573 m”; 1 ♂, 1 ♀, same data except “16°39'42.5"N, 94°13'15.1"W, 1553 m”; 1 ♂, same data except “16°39'36.8"N, 94°13'29.8"W, 1636 m”; 1 ♂, same data except “16°39'36.7"N, 94°13'13.1"W, 1647 m”; 2 ♂♂, 1 ♀, same data except “03-VII-2017, 16°39'44.5"N, 94°13'14.6"W, light trap UV and metallic additives, 1550 m”, J.L. Sánchez-Huerta and V. Moctezuma cols.”; 1 ♀, labeled “MEXICO, Oaxaca, San Miguel Chimalapa, Benito Juárez, 8-X-2015, coprotrap, 16°43'58.4"N, 94°12'17"W, cloud forest, 1356 m, V. Moctezuma col.”; 1 ♀, same data except “16°44'6.2"N, 94°12'20.6"O, 1374 m”; 5 ♂♂, 7 ♀♀, same data except “16°44'10.9"N, 94°12'17.5"W, 1389 m”; 5 ♀♀, same data except “16°44'5.9"N, 94°12'20.5"W, 1384 m”; 5 ♀♀, same data except “16°43'58.6"N, 94°12'22.8"W, 1341 m”; 3 ♀♀, same data except “16°44'12.3"N, 94°12'17.7"W, 1400 m”; 1 ♂, 3 ♀♀, same data except “16°44'10.9"N, 94°12'17.5"W, 1389 m”; 1 ♂, 3 ♀♀, same data except “16°44'7.1"N, 94°12'20.4"W, 1373 m”; 1 ♀, same data except “16°44'12.3"N, 94°12'17.7"W, 1400 m”; 1 ♂, 1 ♀, same data except “16°44'2.4"N, 94°12'20.4"W, 1372 m”; 3 ♂♂, 11 ♀♀, same data except “16°43'53.9"N, 94°12'21"W, 1338 m”; 1 ♂, 1 ♀, same data except “16°44'13.6"N, 94°12'18.1"W, 1405 m”; 2 ♀♀, same data except “16°43'54"N, 94°12'18.4"W, 1347 m”; 1 ♀, same data except “16°43'57.3"N, 94°12'23"W, 1344 m”; 1 ♀, same data except “16°44'23.3"N, 94°12'19.3"W, 1275 m”; 2 ♂♂, 4 ♀♀, same data except “16°43'59.4"N, 94°12'22.3"W, 1342 m”; 19 ♂♂, 16 ♀♀, same data except “16°44'30.6"N, 94°12'20"W, 1269 m”; 1 ♂ same data except “16°44'22.1"N, 94°12'19.9"W, 1293 m”; 2 ♀♀, same data except “16°44'0.5"N, 94°12'22.1"W, 1360 m”; 3 ♀♀, same data except “16°44'4.8"N, 94°12'19.9"W, 1382 m”; 19 ♂♂, 34 ♀♀, same data except “16°44'29.3"N, 94°12'20.1"W, 1275 m”; 9 ♂♂, 8 ♀♀, same data except “16°44'28.1"N, 94°12'20.9"W, 1292 m”; 1 ♀, same data except “9-X-2015, 16°44'22.5"N, 94°13'4.8"W, 1173 m”; 1 ♂, same data except “16°44'21.9"N, 94°13'1.9"W, 1140 m”; 2 ♂♂, 2 ♀♀, same data except “16°44'35.6"N, 94°13'17.5"W, 1113 m”; 3 ♀♀, same data except “16°44'17.1"N, 94°13'4.5"W, 1175 m”; 2 ♂♂, 1 ♀, same data except “16°44'15.8"N, 94°13'4.1"W, 1180 m”; 1 ♂, same data except “16°44'40.7"N, 94°13'17.4"W, 1110 m”; 2 ♂♂, same data except “16°44'26.9"N, 94°13'9"W, 1130 m”; 1 ♀, same data except “16°39'40.1"N, 94°13'22.4"W, 1599 m”; 1 ♂, 1 ♀, same data except “16°44'36.9"N, 94°13'17.3"W, 1112 m”; 1 ♂, 2 ♀♀, same data except “16°44'22"N, 94°13'3.3"W, 1152 m”; 1 ♀, same data except “16°44'14.6"N, 94°13'4.7"W, 1190 m”; 2 ♂♂, 4 ♀♀, same data except “16°44'11.5"N, 94°13'6.5"W, 1204 m”; 1 ♂, 1 ♀, same data except “16°44'16.3"N, 94°13'16.3"W, 1112 m”; 1 ♂, 2 ♀♀, same data except “16°44'27.1"N, 94°13'11.1"W, 1137 m”; 1 ♂, 1 ♀, same data except “16°44'27.3"N, 94°13'12.5"W, 1133 m”; 1 ♀, labeled “MEXICO, Oaxaca, Santa María Chimalapa, Cerro Azul, 19-X-2015, coprotrap, 16°51'37.9"N, 94°43'32.5"W, subtropical rainforest, 382 m, V. Moctezuma col.”; 1 ♀, same data except “16°51'32.8"N, 94°43'30.8"W, 392 m”.

#### Type deposition.

Holotype and 10 ♂♂, 10 ♀♀ paratypes will be deposited in IEXA. Paratypes will be deposited in the following collections: CEMT (10 ♂♂, 10 ♀♀), CMNC (10 ♂♂, 10 ♀♀), CNIN (10 ♂♂, 10 ♀♀), FSCA (10 ♂♂, 10 ♀♀), JLSHC (10 ♂, 10 ♀), TAMU (10 ♂♂, 10 ♀♀), VMC (rest of paratypes).

#### Description.

Holotype male, total length 7.9 mm, maximum elytral width 4.5 mm. Body elongate-oval and convex, dorsum and venter glossy black with cupreous red and green sheen, clypeal margin dark reddish brown. Clypeal margin slightly V-shaped, with rounded teeth, lateral margin of head arcuate. Clypeal surface with coarse and well-spaced punctures, frons surface finely punctate with punctures of regular size, well-impressed clypeogenal suture, genae finely punctate. Pronotum strongly punctate, with coarse punctures of regular size, midline impressed on posterior half of pronotum, anterior pronotal margin incomplete. Pronotal lateral angles acute. Center of pronotal posterior margin with a line of coarse and large punctures. Proepisternum and proepimeron coarsely wrinkled. Elytral surface smooth, shiny, and punctate. Striae well-impressed, becoming deeply impressed on elytral apex. Well-impressed strial punctures, slightly wider than striae. Interstriae flat. Pygidium strongly convex and finely punctate. Pygidium dark reddish brown with green metallic sheen on apex. Protibia tridentate, apical spur expanded into a rectangular shape. Mesotibia with two apical spiniform spurs. Metatibia with apical spur small and spiniform. Ventral apical half of profemur finely punctate, but punctures becoming coarse along posterior margin of profemur. Ventral surface of meso- and metafemur minutely punctate. Internal sac of the aedeagus with two hook-shaped and one filiform copulatrix lamellae; three accessory sclerites (one large canoe-shaped, one small and flat, and one bispiniform); welldeveloped raspule.

**Figure 1.**
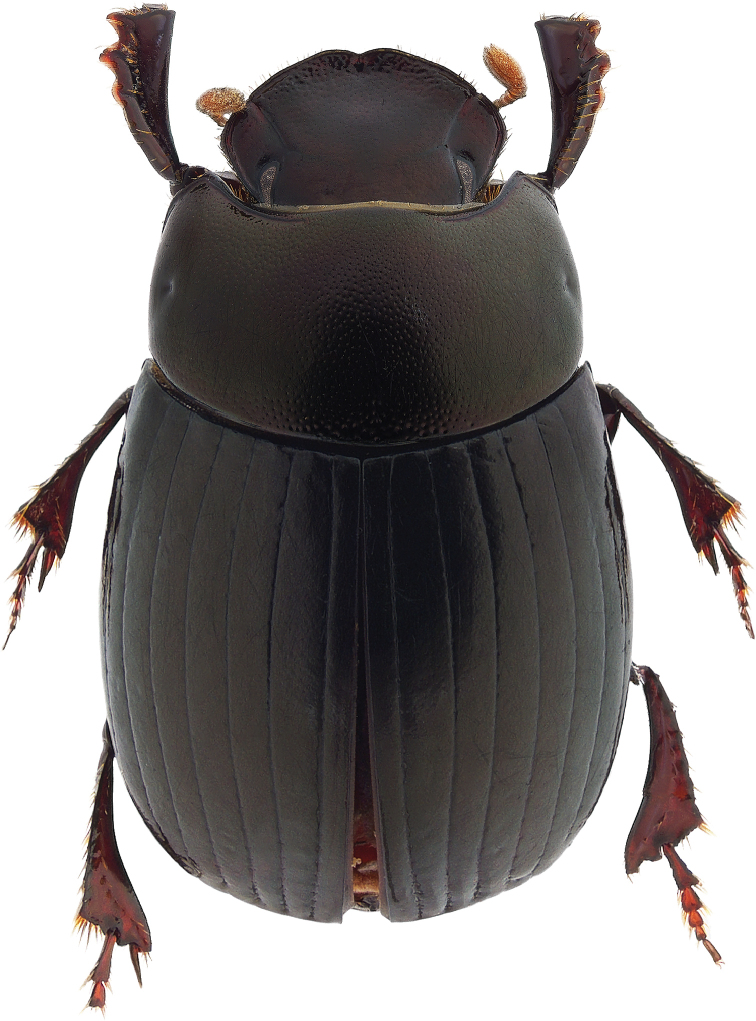
*Ateuchus benitojuarezi*, dorsal habitus.

**Figure 2.**
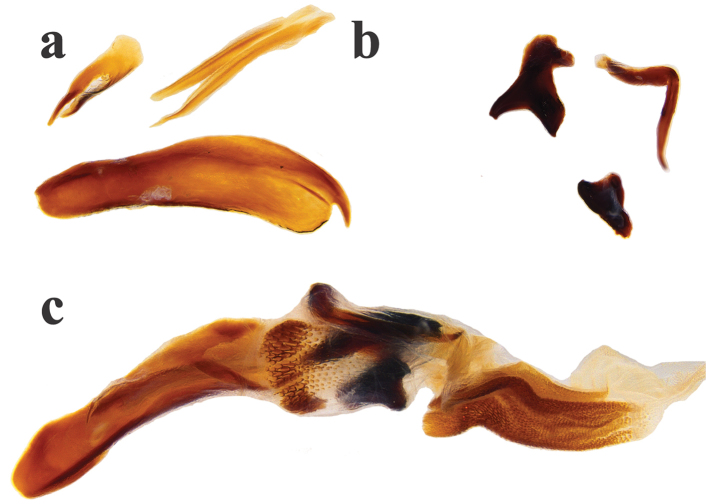
*Ateuchus benitojuarezi*: **a** dissected accessory lamellae **b** dissected copulatrix lamellae and raspule **c** “intact” internal sac of the aedeagus.

**Female.** Differs from the male by protibia with apical spur expanded into a slightly irregular oval shape, clypeal teeth less rounded and more acute, last abdominal sternite broader and pygidium less convex.

#### Variation

(among 30 randomly selected paratypes). Mean total length 8.1 mm (7.5–8.7 mm). Mean elytral width 4.5 mm (4.2–4.9 mm). Type series color is uniform.

#### Etymology.

We dedicate this species to Benito Juárez, the first indigenous president of Mexico. The name also refers to one of the localities where the type series was collected.

#### Geographical distribution.

**Figure 3.**
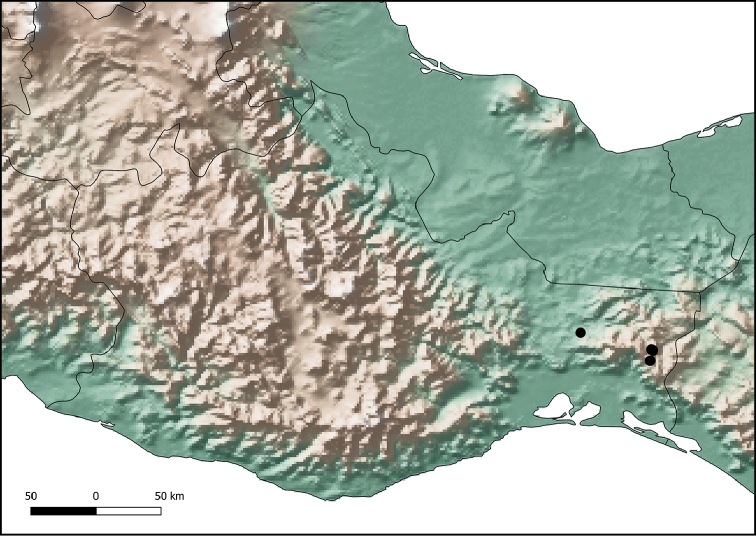
Distribution of *Ateuchus benitojuarezi* (black dots).

The type series was collected in three sites of the region of Los Chimalapas, in the east of the state of Oaxaca: San Antonio, Benito Juárez, and Cerro Azul (Fig. 3).

#### Ecology.

*Ateuchus benitojuarezi* inhabits the cloud forest and the subtropical rainforest, within an elevational range of 380 to 1700 m asl. This species was collected using pitfall traps baited with human dung and with a light trap (mercury vapor and UV light).

#### Diagnosis.

*Ateuchus benitojuarezi* is distinguished from other *Ateuchus* present in Mexico by the following combination of characters: dorsum glossy black with red and green cast, pronotum strongly punctate, anterior margin of pronotum incomplete, posterior pronotal margin with a line of coarse and large punctures, elytral surface smooth and the male genitalia includes one large canoe-shaped lamellae with an apical claw (Fig. 2a).

#### Remarks.

**Figure 4.**
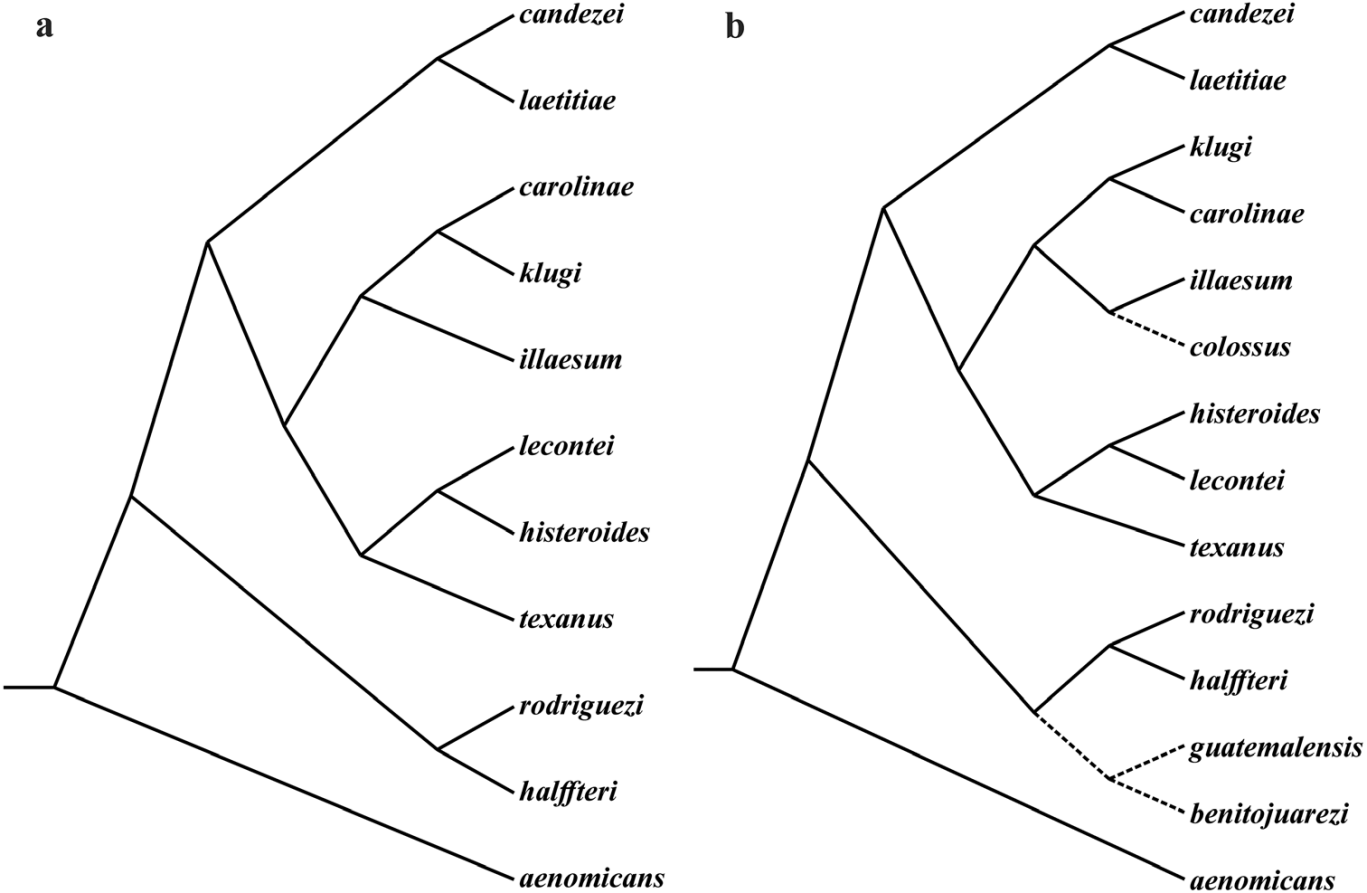
**a** Wagner (cladistic) analysis for *Ateuchus* of North America, based on morphological characters (redrawn from Kohlmann 1984, Kohlmann and Halffter 1988) **b** Same cladogram modified to include putative relationships of *A. benitojuarezi, A. colossus and A. guatemalensis* (dotted lines).

**Figure 5.**
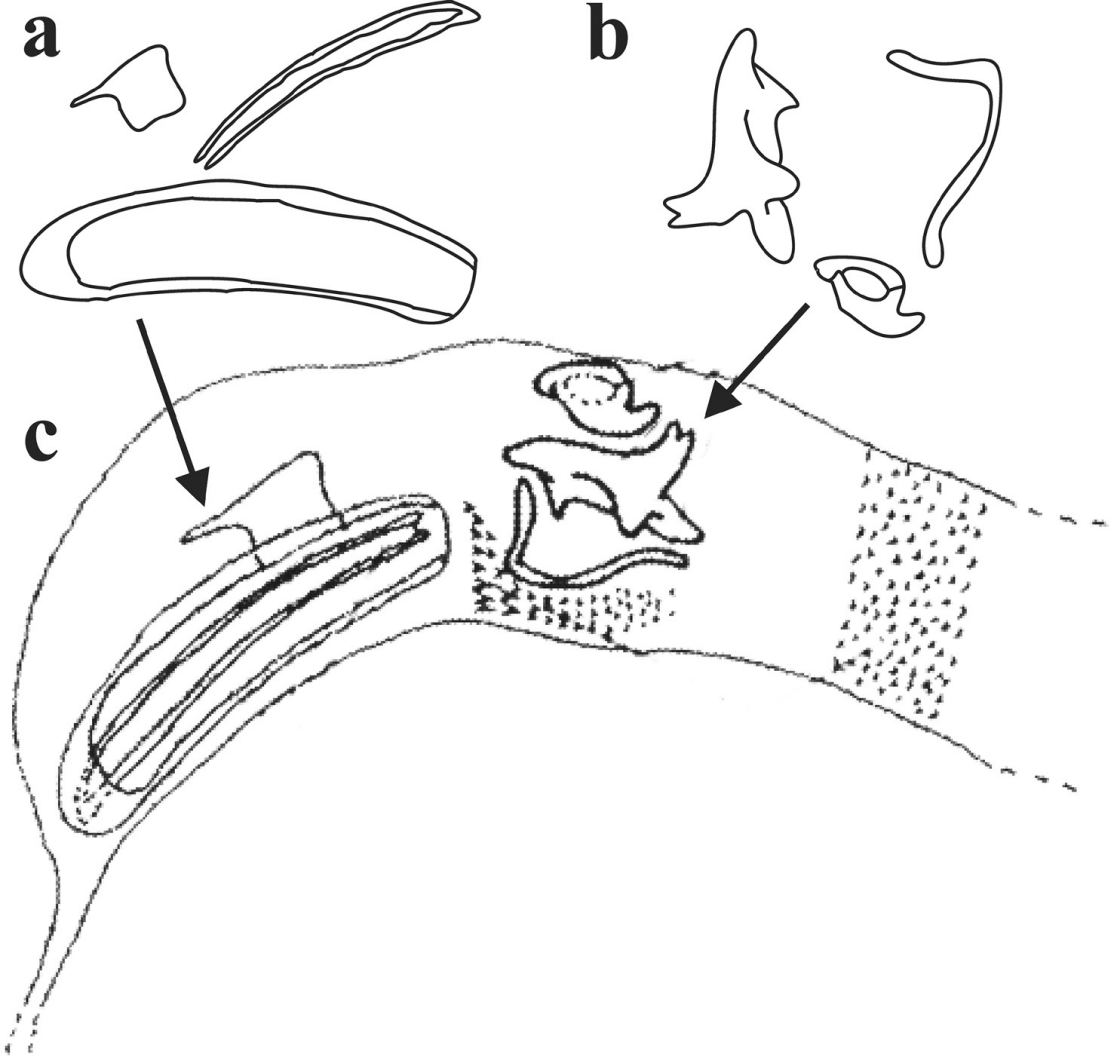
*Ateuchus guatemalensis* (redrawn from Kohlmann 2000, in order to allow comparison with those of *A. benitojuarezi*): **a** dissected accessory lamellae **b** dissected copulatrix lamellae **c** “intact” internal sac of the aedeagus.

*Ateuchus benitojuarezi* seems to be closely related to *A. guatemalensis* (Fig. 4), but the clypeal margin is slightly V-shaped in *benitojuarezi* males (broadly V-shaped in *guatemalensis* males) and the large canoe-shaped accessory lamellae of *benitojuarezi* shows an apical claw (this apical claw is missing in *guatemalensis*; Fig. 5a).

### Ateuchus colossus sp. n.

Taxon classificationAnimaliaColeopteraScarabaeidae

http://zoobank.org/FB4A95B3-55E9-449C-A274-050AC7460511

Figs 6 , 7

#### Type material

(20 ♂♂, 21 ♀♀). Holotype: 1 ♂ labeled “MEXICO, Oaxaca, San Miguel Chimalapa, San Antonio, 14-X-2015, coprotrap, 16°39'39.3"N, 94°13'23.6"W, cloud forest, 1605 m, V. Moctezuma col.”. Paratypes: 1 ♂, same data as holotype; 3 ♀♀ same data except “16°39'40.9"N, 94°13'25.1"W, 1611 m”; 6 ♂♂, 3 ♀♀, same data except “16°39'41.8"N, 94°13'13.9"W, 1548 m”; 1 ♂ same data except “16°39'42.5"N, 94°13'15.1"W, 1553 m”; 5 ♂♂, 5 ♀♀, same data except “16°39'41.2"N, 94°13'15.6"W, 1562 m”; 2 ♂♂, same data except “16°39'36.5"N, 94°13'35.1"W, 1665 m”; 1 ♀, same data except “16°39'40.1"N, 94°13'22.4"W, 1599 m”; 1 ♂, 2 ♀♀, same data except “16°39'40.2’’ N, 94°13'21"W, 1595 m”; 1 ♀, same data except “16°39'40.7’’ N, 94°13'19.7"W, 1593 m”; 1 ♂, same data except “16°39'36.8"N, 94°13'29.8"W, 1636 m”; 2 ♂♂, same data except “16°39'42.5"N, 94°13'15.1"W, 1553 m”; 1 ♀, same data except “16°39'37.5"N, 94°13'27.1"W, 1621 m”; 2 ♀♀, same data except “16°39'41.1"N, 94°13'16.9"W, 1573 m”; 2 ♀♀, labeled “MEXICO, Oaxaca, San Miguel Chimalapa, Benito Juárez, 24-IX-2015, coprotrap, 16°42'38.2"N, 94°6'40.6"W, pine-oak, 1271 m, V. Moctezuma col.”; 1 ♀, labeled “MEXICO, Oaxaca, Santa María Chimalapa, López Portillo, 25-IX-2015, coprotrap, 16°40'28.6"N, 94°3'56.6"W, pine forest, 1219 m, V. Moctezuma col.”.

#### Type deposition.

Holotype and 2 ♂♂, 2 ♀♀ paratypes will be deposited in IEXA. Paratypes will be deposited in the following collections: CEMT (2 ♂♂, 2 ♀♀), CMNC (2 ♂♂, 2 ♀♀), CNIN (2 ♂♂, 2 ♀♀), FSCA (2 ♂♂, 2 ♀♀) JLSHC (2 ♂♂, 2 ♀♀), TAMU (2 ♂♂, 2 ♀♀), VMC (rest of paratypes).

#### Description.

Holotype male, total length 10.7 mm, maximum elytral width 5.6 mm. Body elongate-oval and convex, dorsum and venter glossy black without metallic sheen, clypeal margin dark reddish brown. Clypeal margin slightly V-shaped, with rounded tooth, lateral margin of head arcuate. Clypeal surface with coarse punctures separated by ≈1-2 diameters, frons surface finely punctate with punctures becoming almost unapparent near frons center, well-impressed clypeogenal suture, genae finely punctate. Pronotum smooth but minutely punctate, midline weakly impressed on posterior half of pronotum, anterior pronotal margin complete and well impressed. Anterior pronotal angles very acute and projecting anteriorly. Center of pronotal posterior margin with a line of coarse and well-impressed punctures. Proepisternum coarsely wrinkled, proepimeron finely wrinkled. Elytral surface smooth, shiny, and minutely punctate. Striae well-impressed, becoming deeply impressed on elytral apex. Well-impressed strial punctures, slightly wider than striae. Interstriae flat. Pygidium strongly convex and finely punctate. Pygidium dark reddish brown with green metallic sheen on apex. Protibia tridentate, apical spur expanded into a slightly irregular oval shape. Mesotibia with two apical spiniform spurs. Metatibia with apical spur large and spiniform. Ventral apical half of profemur finely and scarcely punctate, but punctures becoming coarse along posterior margin of profemur. Ventral surface of meso- and metafemur minutely punctate. Internal sac of the aedeagus with three hook-shaped copulatrix lamellae, two small and one large; three accessory lamellae; raspule not well developed.

**Figure 6.**
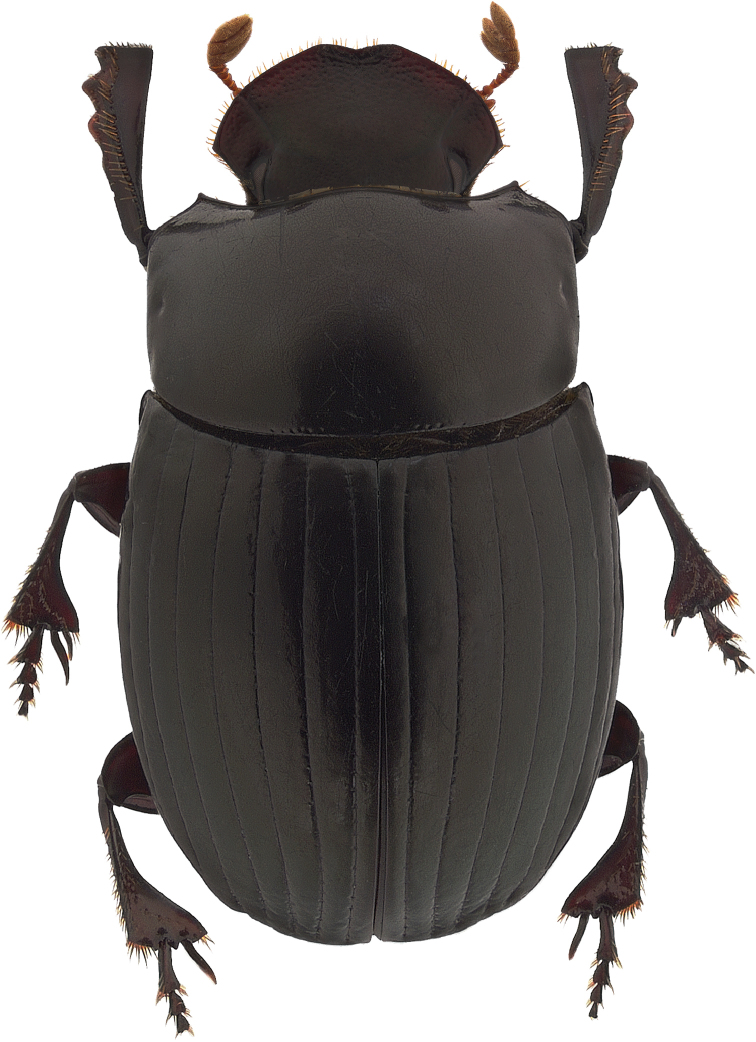
*Ateuchus colossus*, dorsal habitus.

**Figure 7.**
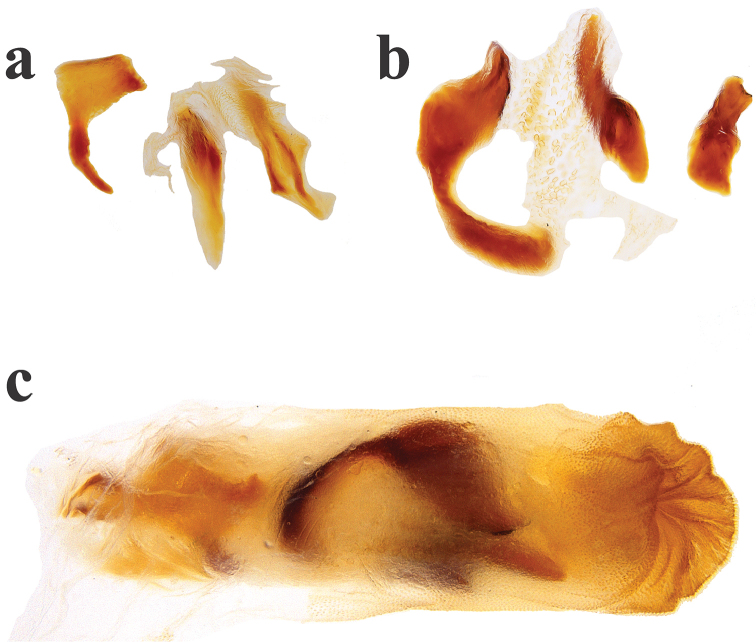
*Ateuchus colossus*: **a** dissected accessory lamellae **b** dissected copulatrix lamellae and raspule **c** intact internal sac of the aedeagus.

**Female.** Differs from the male by coarsely punctate head, protibia with acute and slender spur that bends apically, last abdominal sternite broader and pygidium less convex.

#### Variation

(among 30 randomly selected paratypes). Mean total length 9.9 mm (8.9–10.7 mm). Mean elytral with 5.5 mm (5.2–5.9 mm). Type series color is uniform.

#### Etymology.

From the Latin adjective *colossus* that derives from the Greek noun *κολοσσός* (kolossos = gigantic statue), referring to the fact that this is the largest species of the genus *Ateuchus* in North America known to date.

#### Distribution.

**Figure 8.**
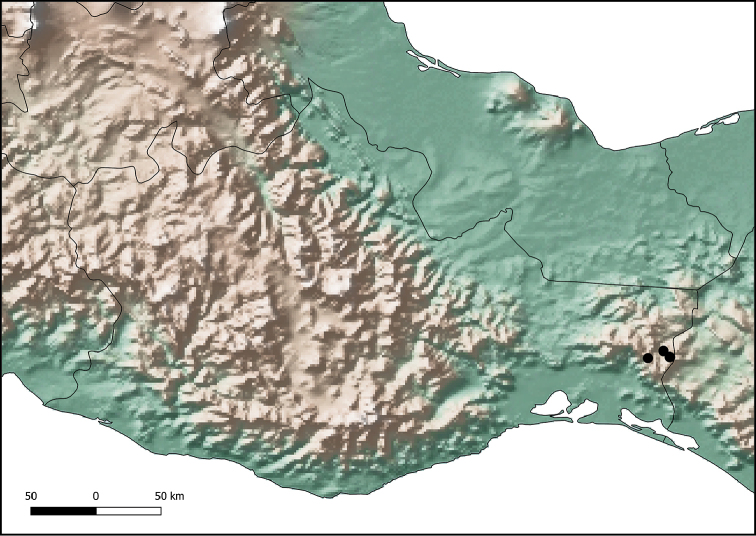
Distribution of *Ateuchus colossus* (black dots).

The type series was collected in the region of Los Chimalapas, in the east of the state of Oaxaca (Fig. 8).

#### Ecology.

This species inhabits cloud and temperate forests (pine and pine-oak), within the elevational range 1219 to 1665 m a.s.l. This species was collected using pitfall traps baited with human excrements.

#### Diagnosis.

This species is distinguished from other North American species by its large body size (at 8.9–10.7 mm, it is the largest *Ateuchus* species in the region), anterior pronotal angles very acute and produced anteriorly, anterior pronotal margin complete, pronotal posterior margin with a line of coarse punctures, and male genitalia with three hook-shaped copulatrix lamellae, three accessory lamellae and ill-developed raspule.

#### Remarks.

**Figure 9.**
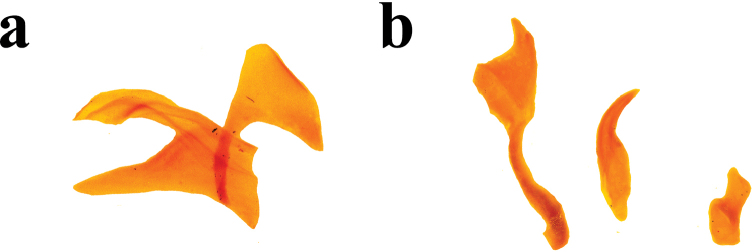
*Ateuchus illaesum*: **a** dissected accessory lamellae **b** dissected copulatrix lamellae.

*Ateuchus colossus* seems to be closely related to *A. illaesum* (Fig. 4), but they can be distinguished by differences in size (*colossus* is larger), the shape of the anterior pronotal angles (very acute and produced anteriorly on *colossus*), accessory and copulatrix lamellae (Figs 7, 9).

## Discussion

The most recent key for identification of *Ateuchus* species of North America was presented by Kohlmann (1984). We decided not to include a key for identification, since a new key for identification is currently in press (Kohlmann and Vaz-de-Mello in press).

We hypothesized that *A. benitojuarezi* is closely related to *A. guatemalensis* and that they represent an evolutionary lineage, since both species present similarities in the general morphology of the sclerites of the internal sac: two hook-shaped and one filiform copulatrix lamellae, and a large canoe-shaped accessory lamella. This combination of shared characters is unique among the known species of North and Central America. Other shared characters of the external morphology are dorsal green sheen, midline impressed on posterior half of pronotum and anterior pronotal margin incomplete. Despite these similarities, *A. benitojuarezi* can be easily distinguished from its sister species by body size (smaller in *guatemalensis*, 5.2–6.7 mm), pronotum punctures, clypeal margin and differences in the sclerites of the internal sac of the aedeagus (Kohlmann 2000).

It has not been possible for us to hypothesize about the evolutionary relationships of *A. colossus* by studying its external morphology. The species of this genus present great morphological homogeneity (Kohlmann 1984). However, the sclerites of the internal sac of the aedeagus of *A. colossus* present similarities to those of *A. illaesum* and therefore these two species could be closely related and form part of an evolutionary lineage. *Ateuchus colossus* possesses notable diagnostic characters that make its identification relatively easy, since it is the largest species of North America and has very acute and apically projected anterior pronotal angles.

In order to determine the evolutionary relationships of the *Ateuchus* species present in North and Central America, it is necessary to conduct new phylogenetic studies. The most recent study that presents a hypothesis of evolutionary relationships is based on a cladistic analysis using morphological characters (Kohlmann 1984, Kohlmann and Halffter 1988). However, the number of species described in the region has since increased. As a consequence of the morphological homogeneity presented by the genus *Ateuchus*, future implementation of molecular phylogenetic analysis would be of great interest.

The genus *Ateuchus* is Neotropical in origin, and is thought to have extended northwards from South America, following the low tropical areas, at the time at which the Panama land bridge was reestablished (Kohlmann and Halffter 1988, Morrone and Halffter 2017). However, the exact period during which the closing of Central America permitted the migration of *Ateuchus* (Pliocene *vs.* Middle Miocene), as well as other groups of organisms, is still the subject of debate (Montes et al. 2015, O’Dea et al. 2016). On entering the Mexican Transition Zone, *Ateuchus* derived in at least four evolutionary lines that coincide with distinct geographic zones: The Pacific slope (*halffteri-rodriguezi*), Mesoamerica (*candezei*-*laetitiae*), the mountains of central Mexico (*carolinae-illaesum*-*klugi*) and the Gulf of Mexico coastal plain (*histeroides*-*lecontei*-*texanus*; Kohlmann 1984, Kohlmann and Halffter 1988).

*Ateuchus guatemalensis* was not included in the cladistic analysis of the *Ateuchus* species of Mexico (Kohlmann 1984). Kohlmann (1984) states that this species corresponds to the phyletic line of the Pacific. Due to the fact that the lineage of the Pacific lacks the putative synapomorphies of *benitojuarezi*-*guatemalensis* (a filiform copulatrix lamellae and a large canoe-shaped accessory lamellae), we hypothesized that they correspond to distinct but closely related lineages.

*Ateuchus benitojuarezi* (Los Chimalapas, cloud forest and subtropical rainforest) and *A. guatemalensis* (Sierra Madre de Chiapas and Central American Mountain Nucleus, cloud forest) fit with the Mountain Mesoamerican distribution pattern (Kohlmann 2000, Halffter and Morrone 2017). This distribution pattern corresponds to typical taxa of the mountain forests of Central America and the south of Mexico (cloud and pine-oak forests), which evolved in the Central American Mountain Nucleus and have ancient South American affinities (Halffter and Morrone 2017). Taking its geographic distribution and ecological affinities as a reference, *A. colossus* also fits with the Mountain Mesoamerican distribution pattern.

Kohlmann and Halffter (1988) state that the distribution of the genus *Ateuchus* corresponds to the Typical Neotropical distribution pattern, although they recognize a similarity with the distribution of various species with the Mountain Mesoamerican distribution pattern. In that study, these authors argue that the penetration of *Ateuchus* into the Central American Mountain Nucleus corresponds to the Pliocene-Pleistocene period. It is not possible to temporally calibrate the cladistic analysis on which they base their conclusions, for which reason this assumption has not been corroborated. It is therefore necessary to conduct molecular phylogenetic or phylogeographic analyses in order to estimate the geologic time in which the penetration and radiation of *Ateuchus* took place in the Mexican Transition Zone, and thus adequately determine the biogeographic affinities of the species of the genus.

In a preliminary manner, we propose the following species as members of the Mountain Mesoamerican distribution pattern (Kohlmann 1981, 1984, 1996–1997, 2000, 2003, Kohlmann and Halffter 1988): *A. carolinae* (Trans-Mexican Volcanic Belt and Sierra Madre del Sur; oak forest and tropical dry forest), *A. fetteri* Kohlmann (Costa Rican Pacific mountains slope, cloud forest and tropical rainforest), *A. gershensoni* (Sierra Madre de Chiapas, cloud forest), *A. ginae* Kohlmann (Central American mountains Pacific slope, cloud forest), *A. hendrichsi* Kohlmann (Costa Rican Pacific mountains, cloud forest and subtropical rainforest), *A. illaesum* (Sierra Madre Oriental and Gulf of Mexico slope; cloud forest, tropical forest, temperate pine-oak and oak forest), *A. klugi* (Trans-Mexican Volcanic Belt and Gulf of Mexico slope; tropical forest, temperate pine-oak and oak forest), and *A. zoebischi* (Costa Rican Atlantic Volcanic slopes, cloud forest and subtropical rainforest).

Little information is available regarding the geographic area of distribution of *A. benitojuarezi* and *A. colossus*. To date, these species have only been collected in the region of Los Chimalapas, in the east of the state of Oaxaca. However, given the habitat preferences of these two new species, they may also be distributed in the states of Chiapas and Veracruz. The two new species are found in sympatry with *A. candezei*, *A. rodriguezi* and an unidentified species of *Ateuchus* (unpublished data).

Little information is available regarding the natural history of *Ateuchus*. They are insects of apparently nocturnal habit, with a broad ecological spectrum. Adults of the genus feed mainly on different types of dung and, to a lesser extent, on carrion, fungi and decomposing plants. Some species present a degree of association with vertebrate burrows and detritus of ants of the genus *Atta* (Navarrete-Heredia 2001, Kohlmann 2003, Génier 2015). To date, we have only suggested the coprophagous habit of *A. benitojuarezi* and *A. colossus*, and a certain degree of attraction to light in *A. benitojuarezi*, since no collections have been made with carrion-baited traps in the region of origin of the two new species. It is unknown whether these species may also be associated with ant detritus. We have not recorded interactions between these species and the burrows of vertebrates; however, in the region of study, pocket gopher burrows are inhabited by *Ateuchus* sp.

Finally, we recommend the use of multifocal pictures in order to illustrate the genitalia and external morphology of the species in future descriptions or redescriptions. We consider it important to illustrate the internal sac of the aedeagus, presenting its components dissected and separated, since this allows greater clarity for comparisons and species identifications. As exemplified in this and previous studies (Moctezuma and Halffter 2017, Génier 2015, Génier and Moretto 2017), this technology allows detailed visualization of the structures of the internal sac of the aedeagus as well as the aedeagus itself, facilitating the identification of taxa of great morphological homogeneity, as is the case of *Ateuchus*.

## Supplementary Material

XML Treatment for Ateuchus benitojuarezi

XML Treatment for Ateuchus colossus

## References

[B1] Halffter G Morrone JJ (2017) An analytical review of Halffter’s Mexican transition zone, and its relevance for evolutionary biogeography, ecology and biogeographical regionalization. Zootaxa 4226: 1–46. https://doi.org/10.11646/zootaxa.4226.1.1 10.11646/zootaxa.4226.1.1 28187628

[B2] Génier F (2015) *Ateuchus cujuchi* n. sp. a new inquiline species of Scarabaeinae (Coleoptera: Scarabaeidae) from tuco-tuco burrows in Bolivia. Zootaxa 3946: 146–148. https://doi.org/10.11646/zootaxa.3946.1.9 25947680 10.11646/zootaxa.3946.1.9

[B3] Génier F Moretto P (2017) *Digitonthophagus* Balthasar, 1959: taxonomy, systematics, and morphological phylogeny of the genus revealing an African species complex (Coleoptera: Scarabaeidae: Scarabaeinae). Zootaxa 4248: 1–110. https://doi.org/10.11646/zootaxa.4248.1.1 28610037 10.11646/zootaxa.4248.1.1

[B4] Kohlmann B (1981) Nuevas especies de *Ateuchus* de México (Coleoptera: Scarabaeidae). Folia Entomológica Mexicana 49: 71–92.

[B5] Kohlmann B (1984) Biosistemática de las especies norteamericanas del género *Ateuchus* (Coleoptera: Scarabaeidae: Scarabaeinae). Folia Entomológica Mexicana 60: 3–81.

[B6] Kohlmann B (1996–1997) The Costa Rican species of *Ateuchus* (Coleoptera: Scarabaeidae). Revista de Biología Tropical 44/45: 177–192.

[B7] Kohlmann B (2000) New species and distribution records of Mesoamerican *Ateuchus* (Coleoptera: Scarabaeidae). Revista de Biología Tropical 48: 253–246.

[B8] Kohlmann B (2003) Tribu Coprini. In: Morón MA (Ed.) Atlas de los escarabajos de México. Coleoptera: Lamellicornia. Vol. II Familias Scarabaeidae, Trogidae, Passalidae y Lucanidae. Argania Editio, Barcelona, 45–58.

[B9] Kohlmann B Halffter G (1988) Cladistic and biogeographical analysis of *Ateuchus* (Coleoptera: Scarabaeidae) of Mexico and The United States. Folia Entomológica Mexicana 74: 109–130.

[B10] Kohlmann B Vaz-de-Mello FZ (in press) A new key for the species of *Ateuchus* Weber (Coleoptera:Scarabaeide: Scarabaeinae) occurring in Mexico, with description of the first North American inquiline species from a rodent burrow (Rodentia: Geomydae) and new distribution records. Revista Brasileira de Entomologia.

[B11] Marchisio R Zunino M (2012) Il genere *Copris* Müller: tassonomia, filogenesi e note di zoogeografia. Monografía 2. World Biodiversity Association, Verona, 174 pp.

[B12] Moctezuma V Rossini M Zunino M Halffter G (2016) A contribution to the knowledge of the mountain entomofauna of Mexico with a description of two new species of *Onthophagus* Latreille, 1802 (Coleoptera, Scarabaeidae, Scarabaeinae). ZooKeys 572: 23–50. https://doi.org/10.3897/zookeys.572.6763 10.3897/zookeys.572.6763 PMC4843985 28050158

[B13] Montes C Cardona A Jaramillo C Pardo A Silva JC Valencia V Ayala C Pérez-Angel LC Rodriguez-Parra LA Ramirez V Niño H (2015) Middle Miocene closure of the Central American Seaway. Science 348: 226–229. https://doi.org/10.1126/science.aaa2815 25859042 10.1126/science.aaa2815

[B14] Navarrete-Heredia JL (2001) Beetles associated with *Atta* and *Acromyrmex* Ants (Hymenoptera: Formicidae: Attini). Transactions of the American Entomological Society 127: 381–429.

[B15] O’Dea A Lessios HA Coates AG Eytan RI Restrepo-Moreno SA Cione AL Collins LS de Queiroz A Farris DW Norris RD Stallard RF Woodburne MO Aguilera O Aubry MP Berggren WA Budd AF Cozzuol MA Coppard SE Duque-Caro H Finnegan S Gasparini GM Grossman EL Johnson KG Keigwin LD Knowlton N Leigh EG Leopnard-Pingel JS Marko PB Pyenson ND Rachello-Dolmen PG Soibelzon E Soibelzon L Todd JA Vermeij GJ Jackson JBC (2016) Formation of the Isthmus of Panama. Science Advances 2: e1600883. https://doi.org/10.1126/sciadv.1600883 10.1126/sciadv.1600883 PMC4988774 27540590

[B16] Wheeler QD Platnick NI (2000) The phylogenetic species concept (sensu Wheeler and Platnick). In: Wheeler QD Meier R (Eds) Species Concepts and Phylogenetic Theory. A Debate. Columbia University Press, New York, 55–69.

[B17] Zachos FE (2016) Species concepts in biology. Historical development, theoretical foundations and practical relevance. Springer, Cham, 220 pp.

